# Autoimmune Hepatitis Following COVID‐19 Vaccination in a Patient With Ulcerative Colitis: A Case Report

**DOI:** 10.1002/ccr3.72686

**Published:** 2026-05-14

**Authors:** Marwah Algodi, Mohamed Nasser Elshabrawi, Mohamed Abuelazm, Ibrahim Gowaily, Omar Saab, Hatem Eltaly, Constantine Fisher

**Affiliations:** ^1^ Jersey Shore University Medical Center Neptune Township New Jersey USA; ^2^ Faculty of Medicine Port Said University Port Said Egypt; ^3^ Faculty of Medicine Tanta University Tanta Egypt; ^4^ Department of Medicine Creighton University School of Medicine Omaha Nebraska USA; ^5^ New York Presbyterian Queens Hospital Flushing New York USA; ^6^ Cleveland Clinic Main Campus Cleveland Ohio USA

**Keywords:** gastroenterology, hepatology, infection, inflammatory bowel disease, liver

## Abstract

Autoimmune hepatitis (AIH) following COVID‐19 vaccination is a rare adverse event. We present the case of a 55‐year‐old male with a history of ulcerative colitis (UC) who developed AIH 3 weeks after receiving his second dose of the Pfizer–BioNTech mRNA vaccine. He presented with jaundice and significantly elevated liver enzymes. A liver biopsy confirmed the diagnosis of AIH. The patient was successfully treated with corticosteroids and azathioprine, leading to complete clinical and biochemical remission. This case highlights the potential for vaccine‐induced autoimmune phenomena, particularly in individuals with preexisting autoimmune conditions, and underscores the importance of prompt diagnosis and treatment.

## Key Clinical Message

This case describes a patient with ulcerative colitis who developed acute autoimmune hepatitis (AIH) 3 weeks after a COVID‐19 booster. While a causal link cannot be definitively proven, the temporal association highlights the importance of considering AIH in the differential diagnosis of postvaccination liver injury. Prompt diagnosis and treatment with standard immunosuppressive therapy led to complete remission.

## 1 Introduction

The worldwide SARS‐CoV‐2 vaccination program constitutes an unprecedented public health success. Still, this vast vaccination program has been linked to rare instances of autoimmune reactions [1]. These events are proposed to be explained by plausible mechanisms, such as molecular mimicry between viral antigens and host proteins [1, 2] or through a nonspecific bystander activation of the immune system that could potentially unmask a preexisting, subclinical autoimmune or metabolic condition such as mild steatosis or indolent autoimmune hepatitis (AIH). Concurrently, case reports have emerged detailing new‐onset or relapsing AIH following COVID‐19 vaccination [3]. This suggests the hypothesis that those with preexisting autoimmune conditions, such as ulcerative colitis (UC), may have a higher vulnerability to such immune dysregulation [4]. We present the unique case of a patient with a known history of UC who developed AIH after receiving the Pfizer–BioNTech BNT162b2 mRNA vaccine.

## 2 Case History/Examination

A 55‐year‐old Hispanic male with a 10‐year history of UC, which had been well‐controlled for the past 5 years on maintenance therapy with oral sulfasalazine alone and without any recent or chronic use of corticosteroids, presented to the emergency department with a three‐week history of dull right upper quadrant (RUQ) abdominal pain and jaundice. His history was notable for receiving his second dose of the Pfizer–BioNTech SARS‐CoV‐2 vaccine 21 days prior to admission. He did not have liver function tests prior to presentation. He reported no recent travel, occupational exposures, or use of herbal supplements or acetaminophen. On examination, he was jaundiced with mild RUQ tenderness. His vital signs were stable, and his body mass index (BMI) was 30 kg/m^2^.

## 3 Methods (Differential Diagnosis, Investigations, and Treatment)

The initial diagnostic workup included comprehensive laboratory testing, viral serologies, and imaging. Autoantibody testing for antinuclear antibody (ANA) and anti‐smooth muscle antibody (ASMA) was performed using indirect immunofluorescence on HEp‐2 cells, with a positive result defined as a titer of ≥ 1:40. A comprehensive viral hepatitis panel was conducted to rule out infectious etiologies. Imaging included an abdominal ultrasound, MRI, and an endoscopic ultrasound. A liver biopsy was performed, and sections were stained with hematoxylin and eosin (H&E). Based on the clinical and histological findings, the patient was started on a 60 mg daily prednisone taper along with azathioprine for immunosuppressive therapy.

## 4 Conclusion/Results

Initial laboratory investigations revealed a significant hepatocellular injury, with an AST of 1621 U/L and ALT of 1476 U/L, accompanied by a total bilirubin of 6.0 mg/dL and elevated inflammatory markers (CRP 45 mg/L and ESR 65 mm/h). A comprehensive viral hepatitis panel, including tests for Hepatitis A (IgM), Hepatitis B (sAg, core Ab, surface Ab), Hepatitis C (Ab), Hepatitis D (IgG), and Hepatitis E (IgG and IgM), was negative. Autoantibody testing was positive for ANA at a titer of 1:160 and ASMA at a titer of 1:80.

**FIGURE 1:**
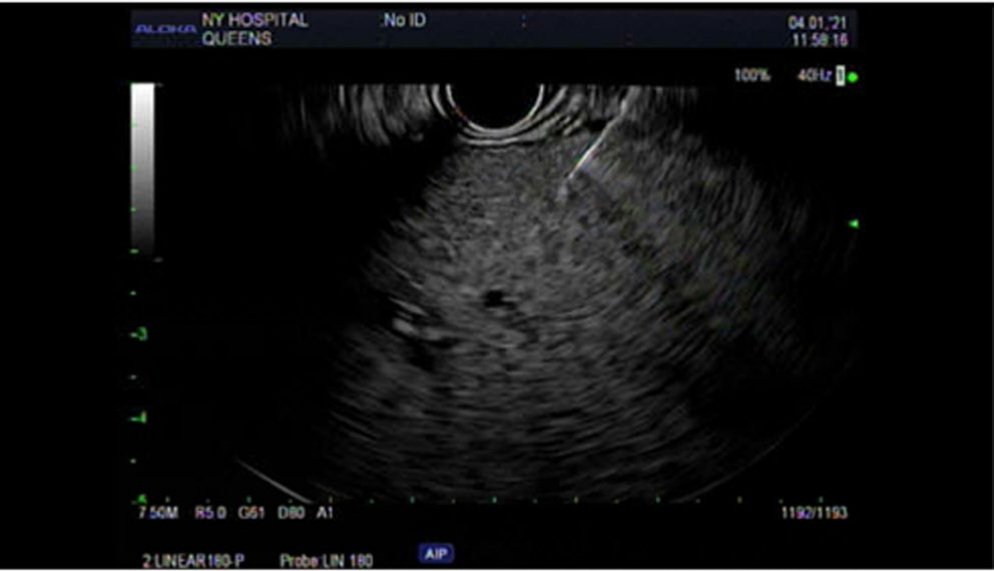
Endoscopic ultrasound view of the liver, displaying a diffuse, abnormal, and heterogeneous echotexture throughout the liver parenchyma, consistent with significant hepatic inflammation.

**FIGURE 2:**
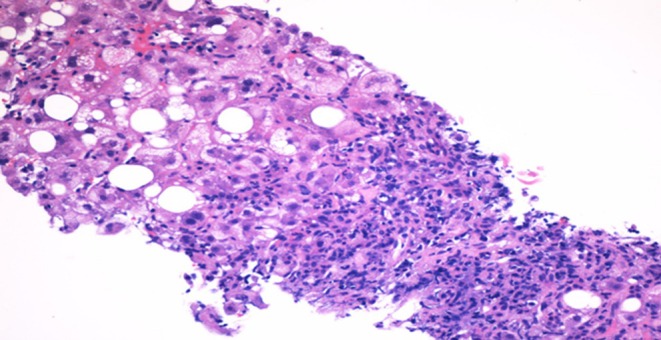
Liver histopathology (hematoxylin and eosin stain), revealing classic features of autoimmune hepatitis, including a dense lymphoplasmacytic infiltrate in the portal tracts with prominent interface hepatitis and evidence of emperipolesis.

While an abdominal ultrasound and MRI were unremarkable, an endoscopic ultrasound showed diffuse abnormal echotexture throughout the liver (Figure 1). A liver biopsy was performed, and sections were stained with H&E (Figure 2). This revealed the classical features of AIH, including a dense lymphoplasmacytic infiltrate in the portal tracts with prominent features, interface hepatitis, and evidence of emperipolesis. In addition, there were clear foci of both macrovesicular and microvesicular steatosis involving approximately 10% of hepatocytes. A trichrome stain for fibrosis showed stage F0.

Based on the clinical picture, timing of vaccination, and exclusion of other causes, a diagnosis of probable vaccine‐associated AIH was made. Following the initiation of immunosuppressive therapy, the patient experienced a complete clinical and biochemical recovery, with liver markers normalizing over 2 months.

Although rare, AIH can follow COVID‐19 vaccination, particularly in predisposed individuals such as those with UC. Early diagnostic consideration and prompt immunosuppressive therapy lead to favorable outcomes. Despite these isolated cases, the risk–benefit ratio overwhelmingly favors vaccination. Continuous reporting and research into these rare autoimmune phenomena will refine our understanding and guide clinical care.

## 5 Discussion

We report a 55‐year‐old Hispanic male with UC who developed AIH 21 days after the second dose of the Pfizer–BioNTech COVID‐19 vaccine. The patient had markedly elevated AST (1621 U/L) and ALT (1476 U/L), positive ANA and ASMA, and experienced a full recovery following the initiation of high‐dose prednisone and azathioprine. This presentation is rare, particularly because it involves UC as a predisposing autoimmune condition, which is an uncommon comorbidity among reported postvaccine AIH cases [4, 5, 6]. While the close temporal association in our case is suggestive, it is critical to acknowledge that the presentation may be coincidental rather than causative. Millions of vaccine doses have been administered, and AIH can occur spontaneously. Establishing true causality is challenging and requires evidence that is often lacking in single case reports.

When evaluating the likelihood of a true causal link, several factors can be considered. First, a rechallenge phenomenon, where hepatitis improves after the first dose and recurs more severely after the second, provides strong evidence. This was not observed in our patient, as he did not have documented liver enzyme monitoring after his first dose. Second, the absence of fibrosis on a liver biopsy would suggest a truly acute process rather than an acute exacerbation of a chronic condition; the trichrome stain showed fibrosis stage F0. Third, the strongest evidence for causality would be sustained remission after withdrawing immunosuppression, a point that requires a long‐term follow‐up beyond our current two‐month window. Finally, while the background annual incidence of AIH in men is low (approximately 1–2 per 100,000), the sheer number of people vaccinated means that some cases of de novo AIH will inevitably occur in the postvaccination period by chance alone. Therefore, while we present a clinically significant case with a striking temporal relationship, we must remain circumspect and consider the association as possibly coincidental.

Proposed mechanisms behind vaccine‐related AIH, if a causal link exists, include molecular mimicry, epitope spreading, and bystander activation, where immune responses to SARS‐CoV‐2 antigens unintentionally target hepatic antigenic structures [6, 7, 8]. An alternative hypothesis is that the vaccine‐induced immune response unmasked a subclinical, undiagnosed mild chronic steatosis or an indolent form of AIH, rather than initiating *de novo* disease from a completely healthy baseline. The finding of microvesicular steatosis on biopsy, while nonspecific, could be compatible with an acute exacerbation of an underlying predisposition. The patient's underlying UC may create a susceptible immunologic environment, predisposing to autoimmune sequelae following vaccination [9].

Most reported AIH cases after COVID‐19 vaccination occurred in middle‐aged women without prior autoimmune diseases, typically 2–6 weeks postvaccination, and presented with features similar to idiopathic AIH, including autoantibodies and a favorable steroid response [5]. AIH following COVID‐19 vaccination has been reported across mRNA, adenoviral‐vector, and inactivated platforms, with onset typically within 1–3 weeks. Most patients were middle‐aged women without prior liver disease, as shown across several reports [10, 11, 12]. However, cases have also emerged in individuals with autoimmune predispositions, such as Hashimoto's thyroiditis and primary sclerosing cholangitis (PSC) [13]. Clinical severity ranged from moderate enzyme elevations to fulminant hepatitis, with one fatal case reported in the Rela case and a patient required a transplant by Efe report [14, 15].

While steroid monotherapy achieved remission in most, several patients required azathioprine in steroid‐refractory disease [13]. Efe et al. further confirmed these patterns in a multicenter cohort, reinforcing the consistency of presentation and treatment response [14]. They report that Pfizer–BioNTech was linked to 59% of post–COVID‐19 vaccine liver injury cases, mostly hepatocellular and immune‐mediated. Most patients recovered, with corticosteroids effective in severe or autoimmune‐like cases. Severe outcomes were extremely rare, with only one requiring liver transplantation [14].

Our case is distinctive for involving a male patient with UC, highlighting the need to monitor vaccine‐triggered AIH not only in women but also in those with preexisting UC. The patient responded promptly to prednisone induction and azathioprine maintenance, consistent with traditional AIH management and outcomes reported in the literature [3]. This suggests that even in vaccine‐triggered cases, standard immunosuppressive regimens are effective.

From a clinical standpoint, this case highlights several important implications. Physicians should maintain a high index of suspicion for AIH in patients presenting with liver dysfunction after vaccination, particularly those with autoimmune comorbidities. Prompt recognition and initiation of corticosteroids remain critical to favorable outcomes. Future research should prioritize systematic surveillance to determine the true incidence of vaccine‐associated AIH, the role of genetic predisposition, and long‐term outcomes across different vaccine platforms. Significantly, these findings should not deter vaccination. The overwhelming evidence continues to support the safety and efficacy of COVID‐19 vaccines, with benefits far outweighing the rare risk of immune‐mediated hepatitis.

This case stands out for its detailed documentation and for highlighting UC as a possible susceptibility factor in vaccine‐associated AIH. It underscores the need for heightened surveillance of liver injury in patients with preexisting autoimmune conditions postvaccination. From now on, prospective studies and registries should aim to clarify incidence, mechanisms, and long‐term outcomes of vaccine‐related AIH.

## Author Contributions

**Marwah Algodi:** conceptualization, investigation, methodology, visualization, writing – original draft, writing – review and editing. **Mohamed Nasser Elshabrawi:** data curation, resources, software, visualization, writing – original draft, writing – review and editing. **Mohamed Abuelazm:** conceptualization, data curation, project administration, supervision, validation, writing – original draft. **Ibrahim Gowaily:** data curation, investigation, methodology, validation, visualization, writing – original draft. **Omar Saab:** data curation, investigation, methodology, validation, visualization, writing – review and editing. **Hatem Eltaly:** conceptualization, data curation, investigation, validation, writing – original draft, writing – review and editing. **Constantine Fisher:** conceptualization, investigation, methodology, project administration, supervision, validation, writing – review and editing.

## Funding

The authors have nothing to report.

## Consent

Written informed consent was obtained from the patient for this case report.

## Conflicts of Interest

The authors declare no conflicts of interest.

## Data Availability

Data can be provided by the corresponding author upon reasonable request.
